# Social robots as skilled ignorant peers for supporting learning

**DOI:** 10.3389/frobt.2024.1385780

**Published:** 2024-08-22

**Authors:** Jauwairia Nasir, Barbara Bruno, Pierre Dillenbourg

**Affiliations:** ^1^ Chair of Human-Centered Artificial Intelligence, University of Augsburg, Augsburg, Germany; ^2^ Computer Human Interaction for Learning and Instruction Lab, École Polytechnique Fédérale de Lausanne (EPFL), Lausanne, Switzerland; ^3^ Socially Assistive Robotics with Artificial Intelligence Lab, Karlsruhe Institute of Technology, Karlsruhe, Germany

**Keywords:** social robots, productive engagement, autonomous social robots, learning companions, educational robots, engagement

## Abstract

When designing social robots for educational settings, there is often an emphasis on domain knowledge. This presents challenges: 1) Either robots must autonomously acquire domain knowledge, a currently unsolved problem in HRI, or 2) the designers provide this knowledge implying re-programming the robot for new contexts. Recent research explores alternative, relatively easier to port, knowledge areas like student rapport, engagement, and synchrony though these constructs are typically treated as the ultimate goals, when the final goal should be students’ learning. Our aim is to propose a shift in how engagement is considered, aligning it naturally with learning. We introduce the notion of a skilled ignorant peer robot: a robot peer that has little to no domain knowledge but possesses knowledge of student behaviours conducive to learning, i.e., behaviours indicative of productive engagement as extracted from student behavioral profiles. We formally investigate how such a robot’s interventions manipulate the children’s engagement conducive to learning. Specifically, we evaluate two versions of the proposed robot, namely, Harry and Hermione, in a user study with 136 students where each version differs in terms of the intervention strategy. Harry focuses on which suggestions to intervene with from a pool of communication, exploration, and reflection inducing suggestions, while Hermione also carefully considers when and why to intervene. While the teams interacting with Harry have higher productive engagement correlated to learning, this engagement is not affected by the robot’s intervention scheme. In contrast, Hermione’s well-timed interventions, deemed more useful, correlate with productive engagement though engagement is not correlated to learning. These results highlight the potential of a social educational robot as a skilled ignorant peer and stress the importance of precisely timing the robot interventions in a learning environment to be able to manipulate moderating variable of interest such as productive engagement.

## 1 Introduction

Social educational robots are gaining momentum because of the advantages and impact they bring over software systems thanks to their physical and social abilities as an embodied agent. Current applications envision the robot to fill roles such as tutor (Kennedy et al., 2016; Ramachandran et al., 2019; Donnermann et al., 2022), peer (Baxter et al., 2017; Kory-Westlund and Breazeal, 2019), mediator (Gillet et al., 2020; Tozadore et al., 2022) or even tutee (Lemaignan et al., 2016; Pareto et al., 2022) and span diverse learning scenarios such as mathematics (Ligthart et al., 2023; Smakman et al., 2021; Hindriks and Liebens, 2019), computational thinking (Nasir et al., 2020; Stower and Kappas, 2021), second language learning (Gordon et al., 2016; van den Berghe et al., 2019) or early language learning (Kanero et al., 2018), story telling (Elgarf et al., 2022b; a) and even handwriting (Chandra et al., 2019; Tozadore et al., 2022).

Simply put, the ultimate aim of social educational robots is to improve the learning gain of the students. The traditional, straightforward approach to achieve this goal is to endow the robots with the domain knowledge needed to understand students’ actions and enhance their learning gain. In contrast to general knowledge, domain knowledge is the knowledge of a specialized field and context. One way for a social robot to acquire domain knowledge is via interacting with its environment through trial and error, e.g., relying on a reinforcement learning framework. While recent advancements in deep learning reassure about its potential for autonomous learning on well-defined problems (Sarker, 2021), enabling robots to autonomously acquire domain knowledge in academic subjects that can be later used in interactions with humans is still an open challenge. Even more challenging is to endow AI/robots with the ability to learn *how* humans acquire knowledge of academic subjects, which is necessary to effectively support human learners in the learning process.

An alternative, more practical but less elegant, method to endow robots with domain knowledge envisions the researchers to simply equip the robot with all the relevant knowledge about the learning problem at hand. This can be done in an offline manner (which is usually the case), for example, in (Ramachandran et al., 2019; Stower and Kappas, 2021; Norman et al., 2022; Ligthart et al., 2023) or in an online manner (Senft et al., 2019). While promising, especially the latter, this solution also presents some drawbacks. In addition to the fact that such robots, by design, are not easily portable from one activity to another without major alterations, the domain knowledge itself is also usually heavily contextualized, typically due to the use of a specific learning platform (Senft et al., 2019; Norman et al., 2022). Hence, the knowledge provided in this manner is typically not only limited to one particular domain, but also one particular learning scenario and paradigm within that domain. As a consequence, manually-programmed social educational robots with only domain knowledge require a significant amount of work, for a limited usability.

Building on research coming out of the learning analytics and psychology communities (Wolters et al., 1996; Fredricks et al., 2004; Kardan and Conati, 2011; Deci, 2017), roboticists are pursuing alternate designs for social educational robots, that seek to bypass the problem of mastering domain knowledge. For example, research has shown that engagement and rapport (particularly during collaborative activities) between the students positively impacts learning (Brown et al., 2013; Chi and Wylie, 2014; Leite et al., 2014; Gordon et al., 2016; Olsen and Finkelstein, 2017; Madaio et al., 2018; Ligthart et al., 2020). Focusing on these constructs thus still allows to follow learning (albeit under an assumption that there is a directly proportional relationship between the modelled construct and learning) without requiring vasts amount of domain knowledge. While these constructs are still known to be influenced, to some extent, by the context, core characteristics could remain indicative over multiple, similar, learning contexts. For example, speech behaviours indicative of conflict resolution and mutual regulation, markers of good collaboration, i.e., collaboration behaviors that help with learning (Blaye, 1988; Schwarz et al., 2000), and in extension markers indicative of student engagement that is conducive to learning in a collaborative setting, can transfer across two collaborative learning tasks with very different underlying learning contexts. Robots relying on these constructs to monitor learning could thus be relatively more versatile and portable.

State-of-the-art methods, typically working *post hoc*, for the modelling of such *domain-agnostic* constructs quite surprisingly do not take the learning gain into account (Lytridis et al., 2020). For example, the very common approach of having expert coders rate synchrony or engagement in pre-recorded data, for example, in (Leite et al., 2015; Engwall et al., 2022), to build a training set for ML methods, typically neglects whether, and to what extent, the students ended up learning and could thus lead to incomplete and possibly misleading notions of engagement, synchrony, etc. This neglect can become especially problematic in open-ended activities where *exploring*, *failing*, *reflecting* and finally *exploiting* the knowledge acquired from all these activities is an essential part of the learning process. In such settings, students’ performance in the learning activity, a more visible phenomenon to the expert coders, does not necessarily translate into their learning (for example, as shown in (Nasir et al., 2020)). A robot tracking and aiming to maximising a construct which is not intrinsically correlated with learning, especially in contexts where the relationship between behaviour and learning is multi-faceted and complex, might thus end up achieving the same performance of one that acts randomly.

In an attempt to contribute to solving this limitation, in previous works we introduced the concept of *Productive Engagement* (PE) (Nasir et al., 2021a; c), an engagement metric that is construed on students’ observable behaviours and positively correlated with learning gain which, in simple words, can be thought of as the *engagement that is conducive to learning*. For this type of engagement, we look at both social and task-related student behaviors, as further explained in (Nasir et al., 2021a) which is a common distinction made in HRI (Corrigan et al., 2013; Oertel et al., 2020) particularly in learning settings (Zaga et al., 2015; de Haas et al., 2022). Building on the concept of *Productive Engagement*, in this paper we propose a robot intervention scheme that tries to reconcile the efficacy of solutions relying only on domain knowledge with the portability of solutions relying on domain-agnostic knowledge.

Such a robot will be able to adequately assess the students’ *Productive Engagement*, on the basis of the students’ learning profiles such as interactive action patterns as well as their speech quantity and quality, and use this information in a way that will allow it to interact with the students in a *helpful-towards-learning* manner. This intervention scheme yields a robot which does not fit in the classical roles of tutor, peer or tutee, which are typically defined on the basis of the different level of domain knowledge the robot possesses.

**FIGURE 1:**
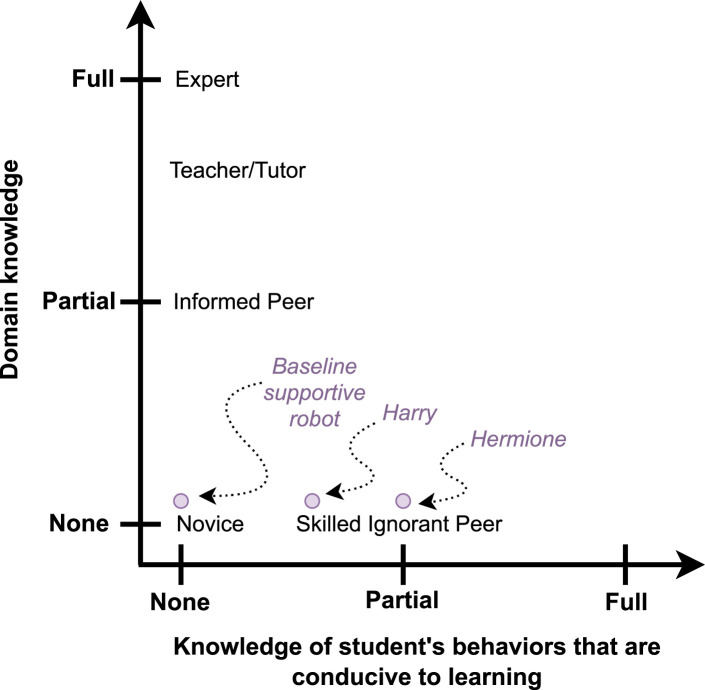
The *skilled ignorant peer* robots *Harry* and *Hermione*, that we propose in this work, placed in the space of domain knowledge and behaviour knowledge. The robot *Harry* knows *what* student behaviours could be conducive to learning, while the robot *Hermione* also knows *when* to intervene to induce the desired behaviours in the learner. Both robots rely on the concept of *Productive Engagement* to assess the learner’s state during the interaction.

A robot relying on *Productive Engagement* (and only on it) to drive its interventions, possesses no direct domain knowledge, and a certain amount of knowledge of the student behaviours conducive to learning: we term such a robot a *skilled ignorant peer*. Concretely, such a robot is clueless as to how to solve a problem, but knows what behaviours are likely to help the students learn, and thus find said solution by themselves. Figure 1 displays the depth of domain knowledge possessed by the robot on the vertical axis and the depth of knowledge it possesses about students’ behaviour that are conducive to learning on the horizontal axis. A number of examples of *skilled ignorant peer* social educational robots can already be found in the literature. One example is a robot that perceives and tries to influence the affective states of the children to provide social support based on the assumption that being in a certain affective state will help improve learning, for example, in a second language learning scenario or a chess playing scenario with children in (Leite et al., 2014; Gordon et al., 2016) respectively, although it is important to notice that the validity of this assumption has not been investigated.

More concretely, the goal of this work is to explore whether *skilled ignorant peer* social robots, relying on the construct of *Productive Engagement*, can be effective for promoting learning. In pursuit of that, we:

• conceptualize the notion of *skilled ignorant peer* social robots that instead of relying on domain knowledge rely on the construct of *Productive Engagement*;

• propose a technical framework for quantifying *Productive Engagement* as PE score

• propose two intervention schemes for *skilled ignorant peer* social robots, one that focuses on the content of the intervention, and another that additionally also carefully chooses the timing of the intervention and then evaluate the two *skilled ignorant peer* social robots in a user study with 136 students;

• propose an experimental method that tries to surface *both* of the following relationships:
  1. Between the robot interventions and *Productive Engagement* (to validate whether the proposed intervention scheme effectively positively influences the students’ behaviour)
  2. Between *Productive Engagement* and the learning gain of the students (to validate whether the students’ behaviour, subject to the real-time interventions of the robot, is still positively correlated with their learning).

To the best of our knowledge, no other work in the literature has formally investigated the two afore-mentioned relationships, i.e., whether or not the robot interventions manipulate the relevant variables of interest which in turn manipulate learning. To better assess the first relationship described above, in this work we propose two *skilled ignorant peer* social educational robots, referred to throughout the article as *Harry* and *Hermione* ^1^.

The reason for designing two robots is the argument that the *timing* of an intervention is as important as the *content* of the intervention, when it comes to nudging students towards behaviours expected to conduce to learning. Hence, the difference between *Harry* and *Hermione* is the fact that the former is equipped with an intervention scheme that knows *what* interventions are likely to positively affect students’ behaviour, but calls them randomly, while the latter is equipped with an intervention scheme that seeks to determine *when* it is best to intervene, and how.

Indeed, previous works in the domain of social educational robots have already emphasized the importance of the appropriate timing of interventions, for example, in the context of suggesting pauses (Ramachandran et al., 2017). The authors compared a robot suggesting children involved in a math activity to make pauses on the basis of a personalization framework with one suggesting to make pauses at fixed intervals and found that the personalized strategy yielded higher learning gains. Then, in (Kennedy et al., 2015), the authors found that caution is needed when designing social behaviors for robots as a too engaging robot, i.e., overly interactive robot, may be distracting for student’s learning gain and eventually counterproductive for the learning process.

In our work, the content of the interventions is shaped by the knowledge on what student behavioural profiles might help in better understanding the learning concepts (that we built in previous works (Nasir et al., 2021c)), while the timing of interventions is shaped by the robot’s ability to detect the absence of the desired student behaviours in real time and trigger the corresponding interventions accordingly. We hypothesize that a robot endowed with this knowledge of *when-and-how-to-intervene* (*Hermione*) will not only promote higher learning gains, but also minimize the disruption of the students’ learning process due to unnecessary interventions, compared to another (*Harry*) which provides randomly picked interventions from the same pool but at fixed times.

## 2 Materials and methods

In this section, we take the reader in-depth into the background, study design and implementation details. The background and study design sections are written such that the readers can go to the results and discussion sections directly if they wish to skip the implementation details.

### 2.1 Background

A previous study we conducted using the baseline version of the same platform that we used in the current study, with 92 children and a baseline robot *Ron* (Nasir et al., 2021c) allowed us to identify three unique sets of student behaviours (in terms of team’s communication behaviour such as speech activity, overlapping or interjecting speech, pauses; their problem solving strategies; facial expressions as well as gaze behaviours), two of which were correlated with higher learning gains. We thus defined: 1) two behavioural profiles for high learning students, respectively denoted as *Expressive Explorers* and *Calm Tinkerers*, which are characterized by different problem solving strategies (global vs. local exploration and reflection) but the same communicative behaviour, involving higher speech activity, many interjections and fewer long pauses; and 2) one behavioural profile associated with low learning students, labelled *Silent Wanderers*, which is characterized by the lack of a clear problem solving strategy, low or no reflection and limited communication, with lower speech activity, few interjections and a higher number of long pauses.

The robot interventions as well as the intervention schemes presented in this paper make use of these findings. More specifically, the aforementioned data corpus, which is publicly available (Nasir et al., 2021b), is used as *training dataset* in this work for modelling the *PE Score*, for generating the problem solving strategy profiles of *Expressive Explorers* and *Calm Tinkerers*, for defining thresholds, for normalizing incoming data, etc. as will be seen in section 2.3.

### 2.2 Study design

#### 2.2.1 Study introduction

##### 2.2.1.1 *JUSThink-Pro*: a collaborative robot mediated game platform

In our study children aged 9–14 interact with *JUSThink-Pro*, an interactive and collaborative human-human-robot game platform for helping to improve computational thinking skills. *JUSThink-Pro* is an extension of the baseline version *JUSThink* (Nasir et al., 2020), which specifically enhances it with (i) the addition of real-time assessment modules that enable the robot to gauge the *Productive Engagement* state of the children and (ii) the integration of real-time robot interventions, driven by the *Productive Engagement* analysis. The game is designed as a collaborative problem-based learning activity for the learning goal of gaining conceptual understanding about minimum spanning trees^2^ (more details about *JUSThink* are given in (Nasir et al., 2020)).

**FIGURE 2:**
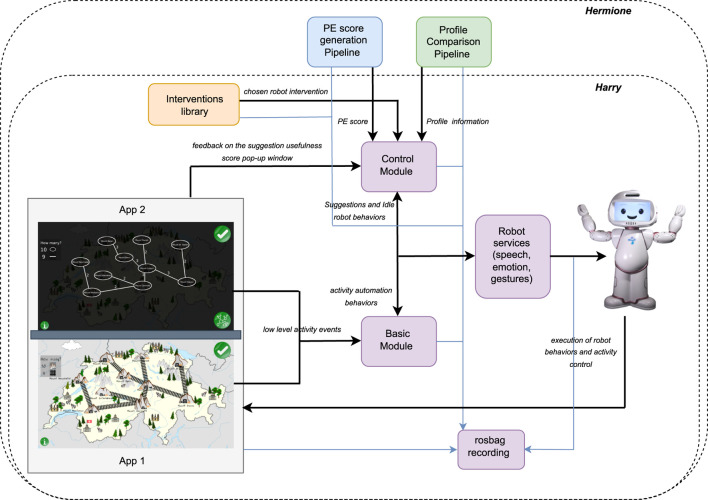
Robot control architecture for *Harry* and *Hermione*.

In this collaborative game, the learning concept is embedded in a fictional scenario set in Switzerland where goldmines, represented as mountains named after Swiss cities, need to be connected by rail-tracks while spending the least amount of money. The game makes players experience two views, with different purposes: the *figurative* view allows a student to interact with the graph and edit (add or remove) tracks (App one in Figure 2), while the *abstract* view allows a student to see the cost of existing tracks as well as all of the team’s previous solutions (App two in Figure 2). The two views swap every two edits, allowing the two students composing a team to experience them both equally. A team is allowed to build and submit as many solutions as they want within the 30 min allocated for the game.

In each session, the robot (*Harry* or *Hermione*) welcomes the team of two students and provides instructions for the session. This is followed by a pre-test that students perform individually. Then, the children collaboratively play *JUSThink-Pro* for 30 min, before concluding with the individual post-test and a questionnaire.

##### 2.2.1.2 Experimental protocol

Our experimental protocol includes two conditions designed to assess the effect of the two robots *Harry* and *Hermione* on the *Productive Engagement* and learning gain of the students. Both robots, using the same *basic module* shown in Figure 2, automate the entire interaction, guide the learners between the various phases of the session, and provide basic motivational feedback as well as the score of each submitted solution. Conversely, the two robots use different *control modules* to generate their real-time interventions. In the case of *Harry*, the *control module* randomly picks at fixed time intervals one among the interventions previously identified (via training data) as correlated with behaviours conducive to learning, from the *interventions library*. In the case of *Hermione*, as shown in Figure 2, the *control module* can rely on additional information about the students provided by the *PE score generation pipeline*, which tracks in real-time the *Productive Engagement* state of the students, and the *profile comparison pipeline*, which monitors their *problem solving strategy*. Concretely, whenever the PE score of a team goes below a certain threshold, *Hermione* picks from the same *interventions library* used by *Harry* the most appropriate intervention considering the phase of the activity as well as the problem solving strategy at the time followed by the team. The *interventions library* includes three types of interventions, namely, *communication inducing*, *exploration inducing*, and *reflection inducing* that are meant to induce, as the names suggest, communication between the two team members, an exploration of the available options or reflections on the current and past solutions, respectively. The design of these interventions, which is further elaborated in Section 2.3, is inspired by the learning profiles and analyses presented in our previous work (Nasir et al., 2021c).

Notice how our *skilled ignorant peer* social educational robots rely on a mix of domain-agnostic information (the PE score and triggering mechanism for robot interventions, as well as the communication-inducing robot interventions) and pseudo-domain-specific information (robot interventions meant to induce exploratory or reflective behaviours that are transversal competencies grounded in the context), and the latter are used within a framework led by the former to support generalizability.

##### 2.2.1.3 Data collection

**FIGURE 3:**
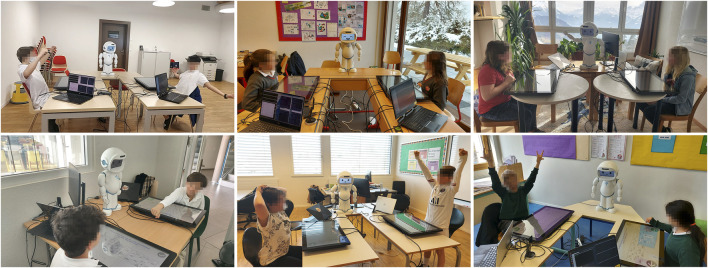
Children interacting with *JUSThink-Pro* at the six schools that participated in the study.

The study^3^ took place over 2 months and involved six private international schools across Switzerland. Figure 3 shows students from the various schools interacting with *JUSThink-Pro*. 136 students (74 male, 62 female) with the age range 9–14 years (median age: 12 years old) interacted with the *JUSThink-Pro* activity for a total of over 70 h in the form of dyads where each dyad interacted only once with the activity for an hour. This gave us a total of 68 teams, out of which some teams were used for validation of the system’s parameters, and due to missing data, two teams were discarded from the experimental set, giving us a total of 52 teams with 26 teams per condition. We have made the data from this study available at (Nasir et al., 2023).

#### 2.2.2 Evaluation metrics

We evaluated the two robot conditions by looking at (i) the students’ learning gains, (ii) their in-task performance, (iii) their PE scores, (iv) their evaluation of the robot’s competence (both during and after the task in the questionnaire).

For the learning gain, we consider the *joint absolute learning gain* (*T_LG_joint_abs*) which is calculated as the difference between the number of questions that both of the team members answered correctly in the post-test and in the pre-test. The reason for using this learning gain is that it captures the shared understanding between the team members that, as established in (Nasir et al., 2021c), is a relevant factor for collaborative learning. The normalized *joint absolute learning gain* ranges between 0 and 1. For the in-task performance, we consider the *last error* of the team, intended as the error of the last submitted solution. In the case a team finds an optimal solution (error = 0) the game stops, therefore making *last error* = 0. The *PE score* is a quantification of the *Productive Engagement* state of the team, computed on the basis of quantifiable observable behaviours found conducive to learning. Further details on the real-time computation of the PE score are given in Section 2.3. To evaluate the students’ perception of the robot competence during the task, whenever a robot intervention is triggered, a pop-up dialogue box appears on the screen of each team member, asking them whether they found the suggestion useful or not. Their joint answers compose a *suggestion usefulness score* with values 1, 0, 0.5 if both found the suggestion useful, not useful, or if they differed in their evaluation, respectively. To evaluate the students’ perception of the robot competence after the end of the task, the students were asked to rate the statements “I think the robot was giving us the right suggestions” (*right suggestions*) and “I think the robot gave us suggestions at the right time” (*right timing*) on a five-points Likert scale.

#### 2.2.3 Hypotheses

Concretely, the study aimed to verify the following hypotheses:

• H1: (a) *Hermione* will lead to higher learning gains as well as (b) a higher number of teams achieving a higher learning gain as compared to *Harry.*
• H2: Teams that interact with *Hermione* will display higher *Productive Engagement* scores compared to the teams that interact with *Harry.*
• H3: *Hermione* will be rated higher than *Harry* on competence.
• H4: Robot interventions will have a positive effect on the *PE score* in both robot conditions.
• H5: Robot interventions will have the desired effect (increase) on learner behaviours in both conditions.
• H6: The *PE score* will be positively correlated with the learning gain in both conditions.

With H1, H2 and H3 we look at general variables of interest such as learning gain, productive engagement, and robot perception and seek to assess the effectiveness of the two proposed intervention schemes for *skilled ignorant peer* social educational robots. H4, H5 and H6 allow for evaluating the two relationships introduced in Section 1: with H4 and H5 we evaluate the relationship between robot interventions (RI) and *productive engagement* (RI to PE), while H6 evaluates the relationship between the PE score and learning gains (PE to LG).

### 2.3 Implementation

#### 2.3.1 Robot interventions design

**FIGURE 4:**
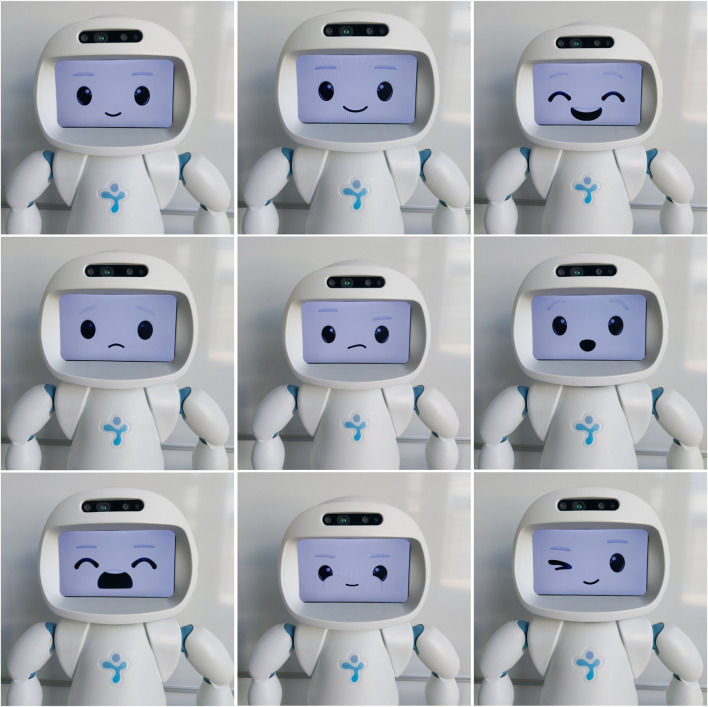
Facial expressions of QTrobot in horizontal order from the top-left corner: neutral, smiling, happy, sad, confused, surprised, bored/yawning, puffing cheeks/being cute, and winking.

Each robot intervention is comprised of verbal and non-verbal components, where the non-verbal component consists of gestures and facial expressions (some of them are shown in Figure 4). Each intervention is designed to induce one of the behaviours that have been found to be conducive to learning in this activity (i.e., that were displayed by teams labelled as *Expressive Explorers* and *Calm Tinkerers*, see (Nasir et al., 2021c) for more details). Specifically, the interventions can be categorized into the following three types:

1. **Exploration inducing:** these interventions seek to induce the behaviour of *Edge Addition* in the learners, i.e., nudge them towards exploring different options to connect goldmines to one another.
2. **Reflection inducing:** these interventions seek to induce the behaviours of *Edge Deletion*, *History* (check previous solutions), *A_A_add* (add back an edge immediately after deleting it), *A_A_delete* (delete an edge immediately after adding it), *A_B_add* (add back an edge immediately after your team member deleted it), and *A_B_delete* (delete an edge immediately after your team member added it). All such actions imply some form of reflection, on the current solution or on the comparison with previous actions and solutions (Nasir et al., 2021c).
3. **Communication inducing:** these interventions seek to induce *Speech Activity* and generally communication between the team members.

It must be noted that, while interventions were designed to explicitly elicit one particular student behaviour, it is not impossible that they also, implicitly induce other behaviours. For example, *Exploration inducing* and *Reflection inducing* interventions can indirectly induce an increase in communication between the team members, as a by-product of attracting students’ attention towards a certain action. Similarly, *Communication inducing* interventions can indirectly induce exploration or reflection actions, as the students share their ideas concerning the next steps or their understanding of the problem. Please note that the robot does not have any idea about what the correct solution is or what would be the best next action to take. Lastly, the style in which each suggestion is conveyed is always supportive and positive. A few examples of interventions are shown in Table 1.

**TABLE 1:** Examples of robot interventions.

Type	Robot’s speech	Facial expression	Gesture
Exploration Inducing	Guys, we may not be exploring all the rail-tracks. Why don’t we connect more gold mines to see how much they cost?	Puffing its cheeks	Putting both arms ahead to gesture while moving head side to side to look at both learners
Exploration Inducing	Are there some tracks we have not explored yet? If yes, why don’t we explore other tracks too?	Smile	Moving head side to side to convey looking at both learners
Reflection Inducing (*Edge Deletion*)	Guys! I have this idea. Why don’t we remove the rail-tracks we don’t need? What do you think?	Puffing its cheeks	Moving head side to side while swiping its arm from left to right
Reflection Inducing (*History*)	Oh hey, may be we have already explored some of these rail-tracks. Should we check our history? I think we did not look at it much in the last few minutes	Smile	Moving head side to side while pointing at the front
Reflection Inducing (*A_A_add*)	Guys, I am a bit lost. I would like you to tell me why is it that you removed the last rail-track?	Confused	Moving head side to side while putting its arms at the back on the hips
Communication Inducing	So Alice, why don’t you tell us about what you think we need to do, and then Bob, you tell us what you think	Neutral expression	Moving head side to side while swiping the right arm
Communication Inducing	So my friends, based on the last few minutes, I feel like we are not communicating much with each other, and that may be important for us to solve this problem	Confused	Moving head side to side while shifting the left arm in a natural movement

Beside interventions, we also designed a pool of idle, non-verbal robot behaviours, consisting of gestures and facial expressions. These behaviours are randomly triggered every few seconds to give students the feeling of interacting with a lively robot and to provide a more natural feel to the interaction. These behaviours are only executed when no other task of a higher priority is being executed. Examples of idle behaviours include: 1) the robot looking side to side to the two team members, 2) the robot scratching its head, 3) the robot looking confused, 4) the robot folding arms behind its back as if observing the situation.

#### 2.3.2 Productive engagement score

The *productive engagement* score (*PE Score*) is designed as a linear combination of the features that we found to be discriminatory between the high-learning teams (henceforth also referred to as “gainers”) and the low-learning teams (henceforth also referred to as “non-gainers”), described in Section 2.1. Discriminant features are “Speech” (S), “Overlap_to_Speech_Ratio” (SO) and “Long_Pauses” (LP). The *PE Score* is thus computed as a linear combination of these features (with positive or negative sign to ensure that higher values of the *PE Score* correspond to a higher learning gain) and each feature is weighted by a factor proportional to the contribution of that feature to the variance in the training data^4^. The PE score is this defined as shown in Equation 1 below:

$$PE\;Score=S*\frac{\alpha \left(SO\right)+\beta \left(1-LP\right)}{\alpha +\beta }$$ (1)

where β=α/2 as LP contributes half as much as SO to the variance in the data. The signs of SO and LP are due to the fact that high-learning teams are linked to higher amount of SO and lower amount of LP.

**FIGURE 5:**
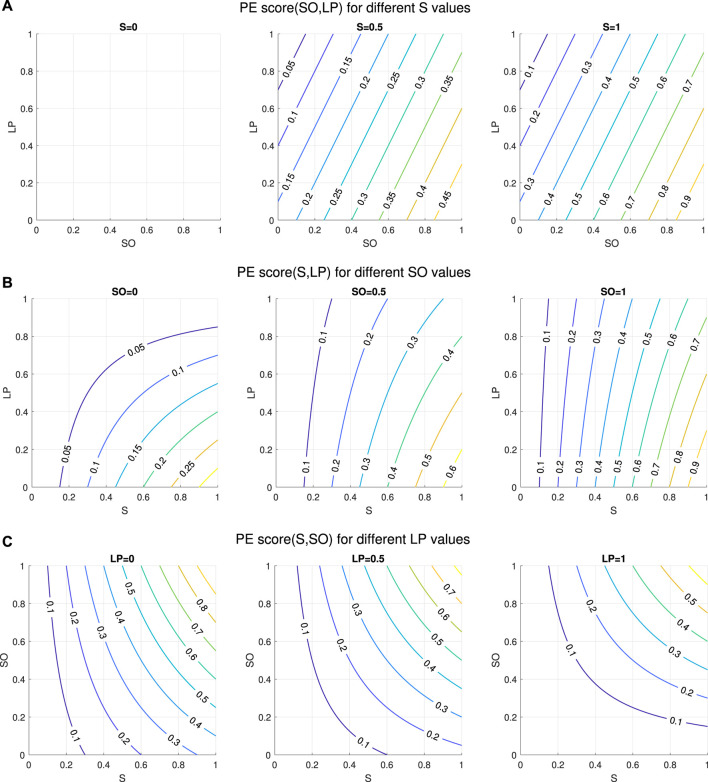
Behaviour of the proposed *PE score* equation. **(A)** *PE Score* when keeping Speech level at 0, 0.5 and 1, respectively. **(B)** *PE Score* when keeping the Overlap_to_Speech_Ratio level at 0, 0.5 and 1, respectively. **(C)** *PE Score* when keeping the Long_Pauses level at 0, 0.5 and 1, respectively.

The *PE Score* can take a value ∈[0,1] . Figure 5 illustrates how our proposed equation for the *PE Score* behaves as a function of its three contributing factors.

To validate the *PE score*, we consider the training dataset and generate the PE score for every 10-s time window for team interactions with the JUSThink game. We perform several tests to verify if the score can be considered a legitimate form of evaluating the *productively engaged* state of the teams, i.e., whether the *PE Score* of high-learning teams is consistently, significantly higher than the *PE Score* of the low-learning teams. To this end, depending if the assumptions for a parametric test are met, we firstly do an unpaired sample *t*-test analysis between the averages of the *PE Scores* and a Wilcoxon rank-sum test between all the points in a *PE Score* sequence for all the high-learning teams *versus* the low-learning teams, both of the tests yield statistically significant differences with *p*-values < 0.01 (details in Table 2). Secondly, we do a Wilcoxon rank-sum test between the Dynamic Time Warping (DTW) distances of every high-learning team 1) with every low-learning team and 2) with every other high-learning team, as well as between the DTW distances of every low-learning team 1) with every high-learning team and 2) with every other low-learning team. Both of these tests yield statistically significant differences with *p*-values < 0.01 (see details in Table 3).

**TABLE 2:** Validation test 1: unpaired sample *t*-test and Wilcoxon rank-sum tests, respectively, for the averages (test 1a) as well as for all the points (test 1b) in PE score sequences of the gainers (G) and non-gainers (NG).

Test 1a			
Group	Mean	Standard deviation	n
G	0.40	0.09	26
NG	0.23	0.03	6
*p*-value = 0.0001			

Test 1b			
Group	Mean	Standard deviation	n
G	0.40	0.18	3,741
NG	0.23	0.15	935
*p*-value = $4.823950e^{-140}$			

**TABLE 3:** Validation test 2: Wilcoxon rank-sum tests between the DTW distances of gainers (G) with the two groups (test 2a) as well as the non-gainers (NG) with the two groups (test 2b).

Test 2a			
Group	Mean	Standard deviation	n
G	1.51	0.46	676
NG	1.69	0.54	156
*p*-value = 0.000134			
Test 2b			
Group	Mean	Standard deviation	n
G	1.69	0.54	156
NG	1.01	0.47	36
*p*-value = $5.897200e^{-15}$			

#### 2.3.3 Generation of the PE score in real-time

**FIGURE 6:**
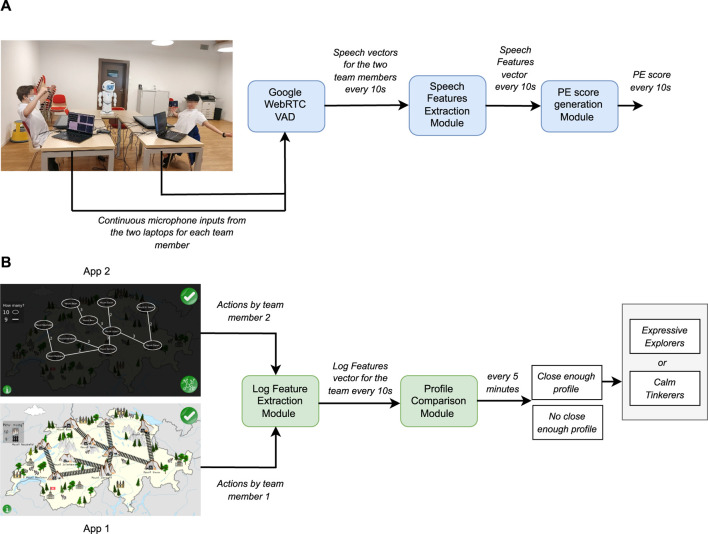
Real time modules for the robot intervention scheme adopted for *Hermione*. **(A)** PE score generation pipeline. **(B)** Profile comparison pipeline.

For the generation of the *PE score* in real-time, we employ the pipeline shown in Figure 6A. We must note that in this pipeline, by real-time, we mean that the *PE score* is updated every 10 s. The audio stream of each team member collected from the laptops’ microphones is analysed by a Voice Activity Detector (VAD) (we use the open-source python wrapper for Google WebRTC VAD^5^). For every 10 s, the VAD returns a vector for each team member that consists of voiced and unvoiced frames (a vector with 0’s and 1’s). These vectors are then used by our feature extraction module to generate the relevant features such as *Speech Activity*, *Speech Overlap*, and *Long Pauses* (see (Nasir et al., 2021a) for details). The features are normalized with respect to the training dataset. Finally, the *PE Score* is calculated with these normalized features as described by Equation 1 discussed above.

As introduced in Section 2.2.1 when describing the intervention scheme of *Hermione*, the *PE score* of a team is compared against a threshold to determine whether an intervention is needed or not. Equation 2 defines how the threshold is generated:

$$\tau_{PE}=\frac{a+b}{2}$$ (2)

where a is the average *PE score* of the high-learning teams in the training dataset, and b is the average *PE score* of the low-learning teams in the training dataset. In our study, the value for $\tau_{PE}$ is set to 0.32.

#### 2.3.4 Profile generation and comparison

The profile comparison pipeline can be seen in Figure 6B. Every time a team member performs an action in the JUSThink activity, the application notifies the log features extraction module. For every 10 s, this module generates all the log-related features of relevance, i.e., those corresponding to the behaviours that the robot interventions seek to trigger, such as *Edge Addition*, *Edge Deletion*, *History*, *A_A_add*, *A_A_delete*, *A_B_add*, *A_B_delete* (see (Nasir et al., 2021b)). The current feature values are then fed to the profile comparison module, that buffers all incoming features until a 5-min timer expires. Then, the module computes the average of each feature until that point in time (on the basis of the values in the buffer and the previous average) and normalizes it with respect to the training data. After that, the module computes the euclidean distance between the normalized log features vector and the reference log features vectors of the *Expressive Explorers* (EE), and *Calm Tinkerers* (CT), generated from our training data. This comparison allows to classify the team’s current problem solving strategy as closer to the global exploratory approach followed by the *Expressive Explorers*, or the local exploratory approach followed by the *Calm Tinkerers*, or neither of the two.

The comparison unfolds as follows. At each time t∈[10,15,20,25] minutes, the euclidean distance $d_{t}^{g}$ of the current feature vector ${cv}_{t}$ of a team from each of the two reference profiles $p_{t}^{g}$ where g∈[EE,CT] is computed, and the lower distance is identified. The current feature vector ${cv}_{t}$ is classified as matching a gainer profile if and only if the lower distance $d_{t}^{g}$ is lower than a threshold $\tau_{t}^{g}$ shown in Equation 3 as:

$$cp_{t}=\;\mathrm{arg min}\;\left[d_{t}^{g}\left(cv_{t},p_{t}^{g}\right)\right]\;⟺\;min\;d_{t}^{g}<\tau_{t}^{g}$$ (3)

In the case that a profile is chosen, the profile comparison module lists the log features from the one in which the incoming vector is farthest to the reference to the one where it is closest. The intuition motivating this choice is that, by triggering a robot intervention aiming to elicit the behaviour corresponding to the farthest feature (i.e., the one that the team is displaying the least), the robot can help the student better align with the problem solving strategy they are already inclining towards, and thus ultimately learn better (the intervention scheme is discussed in detail in Section 2.3.8). In the case none of the distances $d_{t}^{g}$ is *close enough* to the corresponding reference, the robot continues focusing on the communication behaviour of the team.

The thresholds $\tau_{t}^{g}$ are generated *a-priori* on the basis of the training dataset. Specifically, the threshold for each gainer type g∈[EE,CT] is computed according to Equation 4 as:

$$\tau_{t}^{g}=\frac{d_{t}^{\mathrm{intra}}+d_{t}^{\mathrm{inter}}}{2}$$ (4)

where $d_{t}^{\mathrm{intra}}$ is the average intra group (teams belonging to the same learner profile g ) euclidean distance with the centroid profile vector $v_{t}$ for the type of gainers at time t∈[10,15,20,25] minutes and $d_{t}^{\mathrm{inter}}$ is the average inter group (teams within the other gainer group) euclidean distance with the centroid profile vector $v_{t}$ for the type of gainers at time t∈[10,15,20,25] minutes.

The rational for computing the profiles 10 min after the beginning of the interaction, and then every 5 min from then on, stems from an analysis of the training dataset. On those data, we noticed that the profiles generated every 5 min after time t=10 minutes are always consistent with the average profile of that team, over the entire activity. This means that even if within a type of gainer (EE or CT), there are fluctuations over time, when compared with the other profile type at the same time mark, the differences are consistent. For example, the feature of opening up history (*T_hist*) always has a higher value in EE profiles compared to CT profiles at every 5 min mark. However, within the EE profiles at the different time marks, the value for the feature changes.

Let us walk through an example for profile comparison module. At time t=15 minutes, the profile comparison module analyses the feature values it has buffered and computes the distances between the current feature vector of the team and the two reference feature vectors. Let us suppose that the distances $d_{15}^{EE}$ and $d_{15}^{CT}$ of ${cv}_{15}$ are 0.57 and 0.83, respectively. The distance $d_{15}^{EE}=0.57$ is the lowest, and also lower than the threshold $\tau_{15}^{EE}=0.613$ . Hence, the team is classified as adopting a global exploratory strategy and, in case a robot intervention is triggered in the following 5 min, it will take this information into consideration and try to nudge children towards behaviours that are aligned with this strategy.

#### 2.3.5 Validation of the thresholds for the profile comparison module and the PE score module

We used the first 19 teams of the 68 teams that participated in our study for validating the thresholds ( $\tau_{PE}$ , $\tau_{t}^{g}$ for t∈[10,15,20,25] minutes). Please note that five of these teams interacted with *Harry* and were thus kept in the experimental set since the intervention selection scheme of *Harry* does not rely on the thresholds; hence, the validation of these thresholds only matters for *Hermione*. The other 14 teams, that interacted with *Hermione*, were conversely discarded from the experimental data. For $\tau_{PE}$ , we wanted to make sure that the values in the validation data span between the entire range of 0 and 1 as it did in the training data, since the behaviour of the validation data replicated the training data behaviour, the threshold for the *PE Score* was kept as is. For the various $\tau_{t}^{g}$ , we were interested to observe the number of times an incoming profile was detected to be close enough, to ensure that the system was not too strict and never classifying an incoming profile as either *Expressive Explorers* or *Calm Tinkerers*. With the threshold values computed over the training set ( τ for EE at t = 10, 15, 20, and 25 min = 0.532, 0.613, 0.703, and 0.638, respectively; τ for CT at t = 10, 15, 20, and 25 min = 0.818, 0.759, 0.771, and 0.797, respectively), around 30%–40% teams were classified at least once as either of the two gainer profiles. By analysing the teams in the validation set, we decided to increase the thresholds of the type $\tau_{t}^{g}$ by 20% ( τ for EE at t = 10, 15, 20, and 25 min = 0.638, 0.735, 0.843, and 0.765, respectively; τ for CT at t = 10, 15, 20, and 25 min = 0.981, 0.910, 0.925, and 0.956, respectively) to allow for at least 50% of the teams being classified as either of the two gainer type at least once during the course of interaction.

#### 2.3.6 Robot architecture

As shown in Figure 2, the robot control architecture of both *Harry* and *Hermione* includes two modules: 1) a *basic* module, and 2) a *control* module, both sending commands to the robot. The *basic* module is responsible for automating the entire activity and for handling fixed events occurring during the game play, while the *control* module is responsible for the selection and triggering of the robot interventions as well as the idle behaviours during the game play. The whole architecture is implemented in ROS.

The automation of the activity includes tasks such as guiding the team through the different stages of the activity pipeline (explained in Section 2.2.1), explaining what each stage requires the students to do, and giving supportive comments during the game play. Every time a solution is submitted by a team, the *basic* module computes its total cost, which is then verbalized by the robot. In addition to this, the robot randomly reminds the students of the possibility of submitting multiple solutions as well as of the remaining time (the game play is limited to 30 min). The *basic* module is also responsible for pausing the game (i.e., disabling user events) whenever an intervention is triggered by the *control* module, to ensure that students pay attention to the robot. For both *Harry* and *Hermione*, the *basic* module receives information from the two apps as well as from the *control* module and sends commands to the robot via its built-in service controllers. Upon sending a command to the robot, the *basic* module notifies the *control* module, to prevent it from concurrently issuing commands to the robot.

While *Harry* and *Hermione* use exactly the same *basic* module, their *control* modules differ. The *control* module of *Harry* implements the intervention selection Algorithm 1, described below in Section 2.3.7. Conversely, the *control* module of *Hermione* receives information from the *PE score* generation module and the profile comparison module and selects interventions following the algorithm described in Section 2.3.8. The two *control* modules, however, use the same algorithm to generate idle behaviours that is after every 30 s if the robot resources are free, an idle behaviour is executed. For both robots, the *control* module sends commands to the robot via its built-in service controllers and, upon issuing a command, notifies the *basic* module to prevent it from concurrently issuing commands. After every robot intervention i the following metrics, detailed in the following sections, are evaluated and stored: i) the gain in *PE score* $PE_{i}^{\mathrm{gain}}$ ; ii) the associated weight $w_{i}$ ; and iii) the *suggestion_usefulness* score $su_{i}$ . On the basis of the *suggestion_usefulness* score, the robot reacts with comments such as *“Good to know we all agree on the suggestion”* or *“Oh, so you guys do not agree with my suggestion”* before reprising the game play.

#### 2.3.7 Intervention selection scheme of *Harry*

The intervention selection scheme of *Harry*, described in Algorithm 1, is rather straightforward: every time a timer set to rand(0,2) minutes expires (line 4), an intervention is randomly selected from the intervention library and sent for execution to the robot (lines 5–6) and a 2-min countdown is started (line 7). After these 2 min, the timer is reset to a new value rand(0,2) . Concretely, *Harry* will trigger the first intervention within 2 min from the start of the game play, and subsequent interventions with an interval of 2–4 min one from the other. The choice of a gap of a minimum of 2 min between two interventions is arbitrary and meant to let “a reasonable amount of time” pass before another intervention is triggered, to allow to gauge the effectiveness of the intervention.

Lastly, while *Harry* does not make use of the outcomes of the *PE score* generation module and the profile comparison module for the selection of its interventions, their output is still processed and stored for the post-study comparison with *Hermione*.

Algorithm 1 Intervention Selection Scheme for Harry.

1: a= *Exploration inducing* interventions
2: b= *Reflection inducing* interventions
3: c= *Communication inducing* interventions
4:  **Every** rand(0,2) minutes:
5:    Pick an intervention i by rand(a,b,c)

6:    *Harry* executes i

7:    Wait for 2 min
8:    Calculate and store $w_{i}$ , $PE_{i}^{\mathrm{gain}}$ , $su_{i}$

9:    Reset timer

#### 2.3.8 Intervention Selection Scheme for Hermione

Algorithm 2 Intervention Selection Scheme for Hermione.

1: a= *Exploration inducing* interventions
2: b= *Reflection inducing* interventions
3: c= *Communication inducing* interventions
4: ∀i , $w_{i}=0$ , $S_{i}=0$

5:  *EWMA PE Score* = *EWMA of PE Score over a sliding window of 2 min*
6:  **if** *EWMA PE Score* $\geq \tau_{PE}$ **then**
7:   Do nothing
8:  **else if** *EWMA PE Score* $<\tau_{PE}$ **then**
9:   **if** t≤10minutes OR $cp_{t}\neq any\;g\in \left[EE,CT\right]$ **then**
10:    **if** ∀i∈c , $S_{i}=1$ **then**
11:     Sort i based on $w_{i}$ in descending order
12:     Set $S_{i}=0$ for ∀i∈c

13:    **else if** ∀i∈c , $S_{i}\neq 1$ **then**
14:     Pass
15:    **end if**
16:    Pick the first intervention i of type c such that $S_{i}=0$

17:    *Hermione* executes i

18:    Wait for 2 min
19:    Update $w_{i}$ , $PE_{i}^{\mathrm{gain}}$ , $su_{i}$

20:    Set $S_{i}=1$

21:   **else if** t>10minutes AND $cp_{t}=any\;g\in \left[EE,CT\right]$ **then**
22:    **if** ∀i∈a OR b , $S_{i}=1$ **then**
23:     Sort i∈a OR b based on $w_{i}$ in descending order
24:     Set $S_{i}=0$ for ∀i∈a or b

25:    **else if** ∀i∈a AND b , $S_{i}\neq 1$ **then**
26:     Pass
27:    **end if**
28:    Identify the weakest log action based feature of the matched profile
29:    Pick the first corresponding intervention i of type a or b such that $S_{i}=0$

30:    *Hermione* executes i

31:    Wait for 2 min
32:    Update $w_{i}$ , $PE_{i}^{\mathrm{gain}}$ , $su_{i}$

33:    Set $S_{i}=1$

34:   **end if**
35:  **end if**

The intervention selection scheme for *Hermione* is described in Algorithm 2. On the basis of the team’s *PE score*, computed every 10 s by the PE score generation module, the algorithm computes the exponentially weighted moving average (EWMA) of the *PE score*, over a sliding window of 2 min. This value is chosen to ensure that, as in the case of *Harry*, at least 2 min pass in between one intervention and another.

The EWMA *PE score* is then compared to the threshold $\tau_{PE}$ (line 5): if it is above the threshold, the robot does not intervene in order not to disrupt the student’s *Productive Engagement* state (line 6). If the EWMA *PE score* is lower than the threshold, the algorithm considers the phase of the activity and the problem solving strategy adopted by the team to determine the type of intervention to trigger. More precisely, in the first 10 min of game play, or whenever the team’s profile is not close enough to any of the two reference profiles (line 8), *Hermione* picks one of the *communication inducing* behaviour. Conversely, after 10 min of game play, whenever the team’s current profile matches either the *Expressive Explorers* or the *Calm Tinkerers* (line 20), *Hermione* triggers either an *exploration inducing* intervention, or a *reflection inducing* one, on the basis of the weakest log feature returned by the profile comparison module (lines 27–28, see also Section 2.3.4). To avoid having a same intervention (content wise) being triggered multiple times, which could annoy the students, interventions are chosen on the basis of a weighting system. At the beginning of the session, all interventions are assigned a weight of 0 and a flag $S_{i}$ denoting whether they have been triggered is set to FALSE (line 4). Whenever an intervention is triggered, $S_{i}$ is set to TRUE (lines 19, 32) and that intervention is not chosen until all the other interventions associated with the same behaviour have been used too.

Once all interventions associated with the same behaviour have been used (which, however, is quite rare in a 30 min activity), all the flags $S_{i}$ are reset to FALSE. Once the behaviour to induce is identified, among all interventions with $S_{i}=0$ that are associated with that behaviour, the system picks the one with the highest weight $w_{i}$ . The weight of an intervention is updated every time that intervention is triggered as shown in Equations 5, 6 as:

$$w_{i}^{t}=w_{i}^{t-1}+PE_{i}^{\mathrm{gain}}$$ (5)

where

$$PE_{i}^{\mathrm{gain}}=PE_{i}^{\mathrm{after}}-PE_{i}^{\mathrm{before}}$$ (6)

where t denotes time, $w_{i}^{0}$ is the default weight of 0 for intervention i , $PE_{i}^{\mathrm{after}}$ and $PE_{i}^{\mathrm{before}}$ are the values of the *PE score* calculated as an exponentially weighted moving average in the 2 min window after and before an intervention, respectively. The choice of an exponentially weighted moving average in this technique instead of a simple average is to give more weight to the recent quantity and quality of the communication between the team members.

## 3 Results

We first compare the two robot versions in terms of learning gain, productive engagement, and competence (H1-H3) and then we evaluate the two relationships introduced in Section 1: with H4 and H5 we evaluate the relationship between robot interventions (RI) and productive engagement (RI to PE), while H6 evaluates the relationship between the PE score and learning gains (PE to LG). For our analysis, we use two types of tests: Unpaired Sample *t*-test whenever the samples are continuous and satisfy the assumptions for a parametric test, and Wilcoxon rank-sum test whenever at least one sample out of two does not satisfy the assumptions for a parametric test and/or is an ordinal variable.

### 3.1 Comparison between *Harry* and *Hermione* (H1-H3)

**FIGURE 7:**
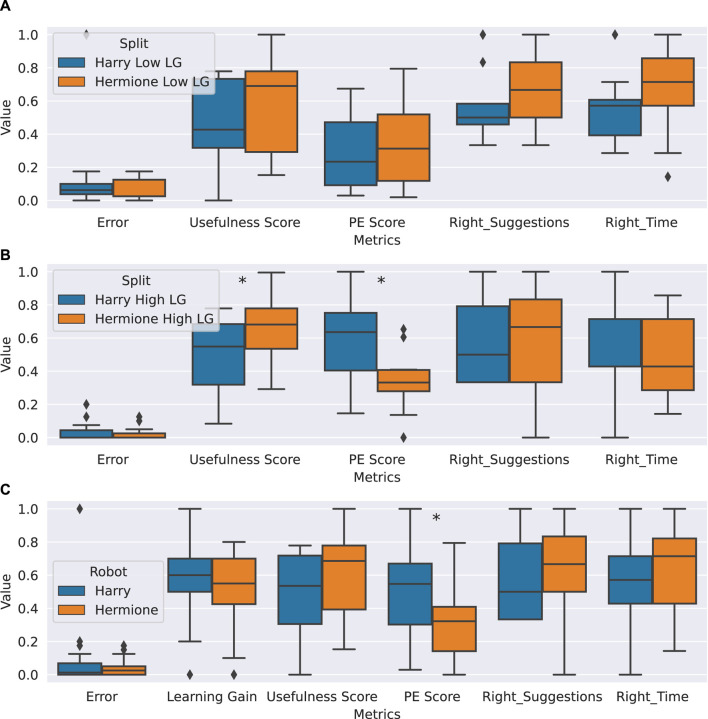
Analysis of hypotheses H1-H3. **(A)** Comparison between the low learning teams interacting with Harry and those interacting with Hermione, on the evaluation metrics presented in Section 2.2.2. None of the metrics differ with statistical significance. **(B)** Comparison between the high learning teams interacting with Harry and those interacting with Hermione, on the evaluation metrics presented in Section 2.2.2. The asterisks on the graph denote significant differences on the statistical test (*PE score*, *p*-value: 0.003) (*suggestion_usefulness* score, *p*-value: 0.05). **(C)** Comparison between the groups interacting with Harry and those interacting with Hermione, on the evaluation metrics presented in Section 2.2.2. The asterisks on the graph denote significant differences on the statistical test (*PE score*, *p*-value: 0.02). For all three sub-graphs, the value on the *y*-axis indicates where the variables on *x*-axis for each of the two conditions lie along the range of 0–1.

To evaluate hypotheses H1-H3, we run a Wilcoxon rank-sum test between the two conditions for the aforementioned evaluation metrics for learning gain, and perceived competence; and an unpaired sample *t*-test for *PE score*. As shown in Figure 7C, there is no significant difference between students interacting with *Harry* and those interacting with *Hermione* in terms of learning gain. Hence, H1(a) is not supported.

Furthermore, contrary to our expectations, the *Productive Engagement* score is significantly higher (*p*-value: 0.02, H: 2.30) for the teams that interacted with *Harry* than those who interacted with *Hermione*. Thus hypothesis H2 is rejected.

Lastly, *Hermione*’s suggestions were preferred over those from *Harry*. The *suggestion usefulness* score is higher for *Hermione* than for *Harry*, with marginal significance (*p*-value: 0.06, H: −1.86 ). Similarly, *Hermione* was rated higher than *Harry* in the questionnaire on both the *right suggestions* and *right timing* items, albeit non-significantly. Hence, hypothesis H3 is partially supported.

#### 3.1.1 High and low learning groups between conditions

In order to investigate H1(b) and better understand the afore-reported outcomes we first verify whether the differences in the *Productive Engagement* score and the *suggestion usefulness* score come from a difference in the number of high and low learning teams in the two conditions.

To this end, we calculate the average learning gain (*T_LG_joint_abs*) of the entire data set (0.559, normalized between 0 and 1) and use a mean split to split the teams in each condition into two groups: one group comprising of teams with high learning gains and the other group comprising of low learning gains. To validate this mean split, we observe via Wilcoxon rank-sum test that indeed the learning gains of the low learning teams are significantly different from the ones of the high learning teams, in both conditions (for *Harry*, *p*-value: $6.33e^{-05}$ , H: −4.00 ; for *Hermione*, *p*-value: $1.46e^{-05}$ , H: −4.33 ). Interestingly, 18 of the 26 teams that interacted with *Harry* ended up with higher learning gains, while only 13 of the 26 teams that interacted with *Hermione* ended up with higher learning gains. Thus, H1(b) is rejected.

Furthermore, we compare the two groups between the two conditions on the evaluation metrics introduced in Section 2.2.2 using Wilcoxon rank-sum tests mainly and unpaired sample *t*-test for the *PE Score*. As Figure 7A shows, there is no difference in terms of any metric between the two groups that have low learning gains, i.e., low learning teams behave similarly irrespective of the robot they interact with. Conversely, significant differences are found when comparing the two groups that have higher learning gains, as shown in Figure 7B, which explain the differences in the *Productive Engagement* score and *suggestion usefulness* score found in Section 3.1. The teams with higher learning gains in the *Harry* condition display a significantly higher PE score (*p*-value: 0.003, H: 3.17) and rate the robot significantly lower on the usefulness of the suggestions (*p*-value: 0.05, H: −1.94 ) as compared to the teams with higher learning gains in the *Hermione* condition.

### 3.2 Correlations between the robot interventions, PE score and learning gain (H4-H6)

**FIGURE 8:**
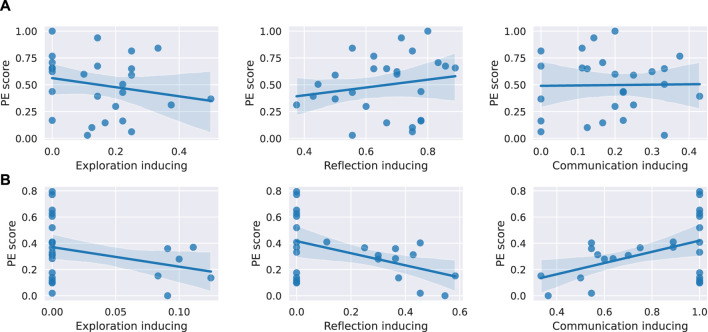
Linear regression between the three intervention types and the *PE score* for the high learning teams in both conditions. **(A)** For high learning teams interacting with *Harry*, none of the intervention types is a statistically significant predictor of the *PE score*. **(B)** For high learning teams interacting with *Hermione*, *communication inducing* and *reflection inducing* interventions are statistically significant predictors of the *PE score* with *p*-values of 0.02 and 0.02, respectively.

To answer the hypothesis H4, exploring the relationship between robot interventions and the PE score, we are first interested in identifying the types of robot interventions that were received by the students in each condition. More specifically, we focus on the high learning teams who interacted with *Harry* and *Hermione*, as it is only between these groups that differences surface in terms of *PE score* and *suggestion_usefulness* score. Indeed, a Wilcoxon rank-sum test reveals that the high learning teams interacting with *Harry* received significantly more *exploration inducing* interventions (*p*-value: 0.05, H: 1.90) as well as *reflection inducing* interventions (*p*-value: 0.002, H: 3.06) compared with the Hermione group while the high learning teams that interacted with *Hermione* received significantly more *communication inducing* interventions (*p*-value: $2.20e^{-05}$ , H: −4.24 ). To verify whether it is indeed the differences in the types of interventions received that causes the observed differences in the PE score between the two groups, we perform a linear regression analysis with the type of interventions as the independent variable and the *PE score* as the dependent variable, for all teams in each of the two groups. We do so by using ordinary least squares (OLS) methods with the statsmodels library (Seabold and Perktold, 2010). As shown in Figure 8A, in the case of the high learning teams interacting with *Harry*, none of the intervention types (*exploration inducing*, *reflection inducing*, *communication inducing*) is a statistically significant predictor of the *PE score* ( β : −0.42 , *p*-value: 0.317; β : 0.36, *p*-value: 0.37; β : 0.03, *p*-value: 0.93, respectively). Conversely, for the high learning teams who interacted with *Hermione* (see Figure 8B), both the intervention types of *reflection inducing* and *communication inducing* are statistically significant predictors of the *PE score* ( β : −0.46 , *p*-value: 0.02; β : 0.42, *p*-value: 0.02, respectively). Unexpectedly, however, they affect the *PE score* in opposite ways. An increase of one in *communication inducing* intervention type seems to be associated with an average increase of 0.42 in the *PE score*, while an increase of one in the *reflection inducing* intervention type seems to be associated with an average decrease of 0.46 in the *PE score*. This finding suggests that interventions, and especially *reflection inducing* ones, require further refinements and testing, to ensure that they all yield a positive effect on the *PE score*. To conclude, H4 is partially supported as some of the interventions have an effect on the *PE score*, although only in the case of *Hermione* and the effect is at times detrimental.

To assess H5, we evaluate the effectiveness of the interventions, i.e., if the corresponding learner behaviour increases in the 2 minutes after the intervention is suggested as compared to the 2 minutes that preceded the intervention. If that is the case, the intervention is considered effective. We can thus compute the percentage of interventions that were effective, for each of the three types of interventions. For both robots *Harry* and *Hermione*, while the *communication inducing* (53% and 48%, respectively) and the *exploration inducing* (42% and 33%, respectively) interventions are effective in a medium range, very few (6% and 10%, respectively) of the *reflection inducing* interventions seem to have been effective. Hence, H5 is only partially supported.

**FIGURE 9:**
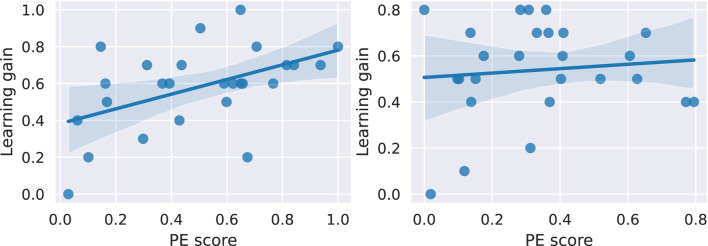
Linear regression between the PE scores and the learning gains of the teams in both conditions. For teams interacting with *Harry* (left), the *PE score* significantly predicts the learning gain with a β of 0.39 and a *p*-value of 0.01. Whereas for teams interacting with *Hermione* (right), we do not find a significant result.

Lastly, hypothesis H6 investigates the relationship between the *Productive Engagement* score and the learning gain *T_LG_joint_abs*. To this end, we again perform a linear regression analysis with the *PE score* as the independent variable and the learning gain as the dependent variable. The results are shown in Figure 9. In the case of teams interacting with *Harry*, the *PE score* significantly predicts the learning gain ( β : 0.39, *p*-value: 0.01) with the fitted regression model as 0.38 + (0.39*PE score), while this is not the case for teams interacting with *Hermione* ( β : 0.09, *p*-value: 0.625). Hence, H6 is only supported for *Harry*.

### 3.3 Summary

Tying our main findings altogether, both robots induce similar learning outcomes (H1a) and similar level of effective interventions (H5), but teams interacting with *Harry* display a significantly higher *PE Score* than those interacting with *Hermione* (H2). For *Harry*, a robot that leverages much less information than *Hermione*, there exists a relationship between the *PE Score* and the learning gain (H6) and a high number of teams (more than in the case of *Hermione*) end up with higher learning gains (H1b). However, the *PE Score* does not seem to be correlated with the interventions of the robot (H4) and the robot’s suggestions are perceived as less useful by the learners (H3). On the contrary, for *Hermione*, there exists a relationship between some of the robot’s interventions and the *PE Score* (H4) and the robot’s suggestions are perceived as more useful by the learners (H3). However, there is no correlation between the *PE Score* and learning gain (H6).

## 4 Discussion

To interpret our results, we go back to the two sides of the equation that links robot interventions to students’ *productive engagement* and students’ *productive engagement* to students’ learning.

In the case of *Harry*, teams received significantly more *reflection inducing* and *exploration inducing* interventions which, although found to induce the desired behaviour 6% and 42% of the times, were not found to impact the *PE score* in any way. For this, we hypothesize that the timing of interventions, not taken into consideration by *Harry*, could be extremely crucial to define this relationship. In turn, the *PE score* was found to be positively correlated with the students’ learning gain. Conversely, in the case of *Hermione*, teams receive significantly more *communication inducing* interventions which elicit the desired behaviours 48% of the times and were found to positively affect the *PE score*. This suggests that a more *conscious* action selection strategy, i.e., that of *Hermione*, can indeed significantly influence the variable of interest *Productive Engagement*, showing the potential of such a *skilled ignorant peer* robot. However, the *PE score* of students interacting with *Hermione* was not found to be correlated with their learning gain. We provide two hypotheses for this: 1) the linear correlation between the *PE score* and the learning gain holds valid only above a given threshold. Indeed, for teams interacting with *Hermione*, the *PE score* is not only significantly lower than the one of those interacting with *Harry*, but generally lying around low values, with a mean value of 0.33 which is very close to the threshold value $\tau_{PE}=0.32$ set to trigger interventions (see Section 2.3.3); 2) the linear correlation between the *PE score* and the learning gain is an insufficient approximation of the real relationship between the two constructs and, specifically, may no longer hold true in case of interventions actively impacting the *PE score* (in the case of *Hermione*).

The analysis of the *reflection inducing* interventions is particularly interesting. While teams that interacted with *Harry* received significantly more interventions of this type than those who interacted with *Hermione*, the former seemed unaffected by the interventions, while the latter saw a decrease in their *PE score*. Considering that, in the case of *Hermione*, the positive effect of *communication inducing* interventions on the *PE score* was counteracted by the negative effect of the *reflection inducing* ones, this clash might be part of the reasons why no conclusions can be drawn on the link between the *PE score* and learning gain in the case of *Hermione*, in addition to the hypotheses mentioned above. The detrimental effect that *reflection inducing* interventions had on the *PE score* in *Hermione* and their general limited effectiveness in inducing the desired behaviours (6% and 10% for *Harry* and *Hermione*, respectively) suggest that future studies should particularly refine the *content* of the *reflection inducing* interventions to successfully induce the desired behaviours.

To summarize, our results with a *skilled ignorant peer* social educational robot demonstrate both theoretical and practical implications that we highlight below.

### 4.1 A perspective shift when modelling subjective constructs

Our modelling of engagement represents a shift towards *aligning* it with the ultimate goal of student learning, which is the cornerstone of the design of any social educational robot. The practical but naive assumption of a linear relationship between engagement (however measured!) and learning, as our study shows, is incomplete at best. A key goal of this work was to better characterize this relationship and better evaluate it, while concurrently assessing the relationship a robot’s interventions have with such engagement.

We contend that this paradigm shift can be regarded as a fundamental *design principle* with profound implications for HRI. It offers guidance for modeling and validating subjective constructs such as engagement, rapport, synchrony, collaboration, etc., in educational human-robot interaction settings, emphasizing that these constructs are not endpoints in themselves but rather integral means to achieve the ultimate educational goal.

### 4.2 Timing matters

One major challenge for social robots in real-world HRI is to initiate communication or provide feedback when it is least disruptive to the interaction at hand. In the specific case of educational social robots, the overarching aspiration is to build a robot that intervenes in a timely manner such that its interventions are least disruptive to the learning process, and most likely to be well received by the students both objectively (as observed in their subsequent behaviour) and subjectively (as rated based on their personal experience). Consequently, our investigation of the effect of robot interventions and their timing on the students’ engagement state, intrinsically tied to learning, and on their perception is a timely contribution. We argue for the inclusion of *validation checks* in HRI studies where robot behaviours are driven by students’ behaviours (here, embedded in *Productive Engagement*) to gauge the extent to which the robot interventions *manipulate* the variables of interest, thereby verifying the reliability of the robot design. For example, in our work, the variable of interest *Productive Engagement* was not manipulated at all by *Harry* but it was manipulated in the case of *Hermione*. Future work can focus on how to leverage this relationship to eventually lead to higher learning.

### 4.3 Outlook

While the paper demonstrates the potential of using a social educational robot as a *skilled ignorant peer* in an educational environment, some limitations need to be highlighted and addressed in future work. As a part of idle behaviours, the robot would randomly sometimes scratch it’s head or look confused which could influence the children’s problem solving behaviour when the team is closer to a solution. While we did not directly observe such a situation, this nonetheless is a possibility; hence more careful design and choice of idle robot behaviours should be considered. Then, since all data used in this work were collected at international schools in Switzerland, they refer to a very specific pool of students, coming from a certain economic and social background; hence, any generalization requires further studies. Furthermore, the training data is not balanced in terms of the two classes of gainers and non-gainers.

Similarly, there is a need to apply the framework of *Productive Engagement*, i.e., the design methodology for an autonomous *skilled ignorant peer* social educational robot equipped with the concept of *Productive Engagement*, in contexts other than the *JUSThink* activity. This would allow us to better understand how the framework generalizes to other tasks and learning activities. For instance, our task relies on a shared visual workspace which has an influence on the possible problem solving strategies and interactions. Other tasks might not have the same characteristics. Therefore, in the future, we would like for this framework to be adopted and evaluated in other HRI learning activities as well as other learning contexts.

## Data Availability

The datasets presented in this study can be found in online repositories. The names of the repository/repositories and accession number(s) can be found in the article/supplementary material.

## Footnotes

1 The names were chosen by the first two authors, who are avid fans of the *Harry Potter* books, in a lively and thoroughly enjoyable discussion. The authors argue that, throughout the book series, Harry displays a rather intuitive understanding of what is the right thing to do and spends little time dwelling onto why it is so. Conversely, the *know-it-all* Hermione always knows what to do, and why, and when. Please note the names are only for us (and the research community hereafter) to refer to different variants. The students are never exposed to the names to avoid any biases. In experimental settings, the robot always introduces itself to all students as *QTrobot*.

2 Let G=(V,E) denote a connected, undirected, edge-weighted graph. V is the set of nodes, E⊆V×V is the set of edges that connects node pairs, and c:E→R is the edge cost function for G . A subgraph of G is said to “span” the graph G if it connects all nodes of G , i.e., each node is reachable from every other. The problem is to find a subgraph T of G that spans G and minimises $o_{T}\left(T\right)=\sum_{e\in E_{T}}c\left(e\right)$ . An optimal solution T is called a minimum spanning tree for G .

3 Ethical approval for this study was obtained from the EPFL Human Research Ethics Committee (051–2019/05.09.2019).

4 Note that a linear combination is one possible way and the most straightforward way but may not be the only way of modelling productive engagement in the form of a PE score.

5
https://webrtc.org/
